# A Narrative Review of The Role of Foods as Dietary Sources of Vitamin D of Ethnic Minority Populations with Darker Skin: The Underestimated Challenge

**DOI:** 10.3390/nu11010081

**Published:** 2019-01-03

**Authors:** Jing Guo, Julie A. Lovegrove, David I. Givens

**Affiliations:** 1 Institute for Food, Nutrition and Health, University of Reading, Reading RG6 6AR, UK; d.i.givens@reading.ac.uk; 2 Hugh Sinclair Unit of Human Nutrition and Institute for Cardiovascular and Metabolic Research, University of Reading, Reading RG6 6AP, UK; j.a.lovegrove@reading.ac.uk

**Keywords:** ethnic, vitamin D intake, vitamin D status, food fortification

## Abstract

In recent years, vitamin D deficiency has attracted attention worldwide. Especially many ethnic minority populations are considered at high-risk of vitamin D deficiency, owing to a lesser ability to synthesis vitamin D from sunlight (ultraviolet B), due to the skin pigment melanin and/or reduced skin exposure due to coverage required by religious and cultural restrictions. Therefore, vitamin D intake from dietary sources has become increasingly important for many ethnic minority populations to achieve adequate vitamin D status compared with the majority of the population. The aim of the study was critically evaluate the vitamin D intake and vitamin D status of the ethnic minority populations with darker skin, and also vitamin D absorption from supplements and ultraviolet B. Pubmed, Embaase and Scopus were searched for articles published up to October 2018. The available evidence showed ethnic minority populations generally have a lower vitamin D status than the majority populations. The main contributory food sources for dietary vitamin D intake were different for ethnic minority populations and majority populations, due to vary dietary patterns. Future strategies to increase dietary vitamin D intake by food fortification or biofortification needs to be explored, not only for the majority population but more specifically for ethnic minority populations who are generally of lower vitamin D status.

## 1. Introduction

Humans can obtain vitamin D both from ultraviolet B (UVB) irradiation [1] and dietary sources [2]. Vitamin D, including cholecalciferol (vitamin D_3_) synthesis in the skin triggered by UVB and vitamin D (vitamin D_2_ and vitamin D_3_) intake from diet (including dietary supplements), needs to undergo two hydroxylation reactions for activation in the human body. The first occurs in the liver where vitamin D is converted to 25-hydroxyvitamin D (25(OH)D) [3], and the second in the kidney to form the physiologically active form vitamin D, 1,25-dihydroxyvitamin D (1,25(OH)_2_D). Serum or plasma 25(OH)D concentration is commonly used as a measure of vitamin D status [3], as it is the main circulating form of vitamin D. Serum or plasma 25(OH)D ≤ 25 nmol/L has been used to define vitamin D deficiency [4] although higher values have been proposed [4].

Vitamin D is a key nutrient for normal bone growth and mineralisation. Recently, mounting evidence shows that low vitamin D status is also associated with increased risk of cardiovascular disease (CVD) and type 2 diabetes (T2D), which are the leading causes of morbidity and mortality in the world [4]. Vitamin D deficiency is prevalent and has become a major health problem globally [5,6]. Especially ethnic minority populations (e.g., Asian, Black) with darker skin are considered as a high-risk group for vitamin D deficiency, owing mainly to having less ability to synthesise vitamin D from sunlight due to the skin pigment melanin and/or overall clothing required by their religion [7]. Furthermore, a resurgence of childhood rickets has recently highlighted the need for adequate vitamin D status [8]. Several environmental factors (e.g., season, latitude, length of day) and personal characteristics (e.g., skin melanin content, ageing) and human behaviour (e.g., sunscreen usage, clothing) limit humans to derive vitamin D from UVB [9]. Therefore, vitamin D intake from the dietary sources has become more important than before for contributing to vitamin D status.

In the current study, ethnic minority populations refer to populations within a community which has different national or cultural traditions from the majority population, and with darker skin. The main objective of the present review is to critically evaluate the vitamin D intake and vitamin D status of ethnic minority populations with darker skin compared with majority populations, and also vitamin D absorption from supplements and UVB of the ethnic minority populations is reviewed. In addition, current strategies of increasing dietary vitamin D for ethnic minority populations is considered. 

## 2. Vitamin D Status and Vitamin D Intake of Ethnic Minority Populations

### 2.1. Methods

A review of the literature on vitamin D status, vitamin D intake in ethnic minority populations was conducted using the online databases PubMed, Embase and Scopus by searching key words of ‘vitamin D status’, ‘vitamin D intake’ and ‘ethnic’. Studies (observational studies, randomized controlled trials (RCT)) published up to October 2018 (without language restriction) were searched. We excluded studies on animals, or populations with a disease or medical condition. In addition, supplementary hand searching of reference lists of previous review was conducted. Total 865 publications were screened for this narrative review. Data were extracted from the studies if vitamin D status were available for both majority population and ethnic minority populations.

### 2.2. Vitamin D Status of Ethnic Minority Populations

Studies reporting vitamin D status in different ethnic minority populations are presented in Table 1 [10,11,12,13,14,15,16,17,18,19,20,21,22,23,24]. The study of Black et al. [12] reported vitamin D status in the West Australian Pregnancy Cohort, which showed the vitamin D status of Caucasians to be significantly higher than non-Caucasians (*p* < 0.001). Results from other studies [13,19,22,24] are consistent with the findings from Black et al. [12]: Cauley et al. [13] reported vitamin D status in White, Black, Asian and American Indian women of the Women’s Health Initiative Observational Study. The vitamin D status of the White groups (60.8 nmol/L) was significantly higher than other ethnicities, and Black populations had the highest number of people (70.5%) whose vitamin D status was lower than 63.6 nmol/L, while White groups have the lowest number of people (30.7%). However, the results of Cauley et al. [13] may be confounded by seasonal variability, as the seasonal variability of the vitamin D status was not controlled. The study of Meyer et al. [19] measured vitamin D status in Norway and showed the mean 25(OH)D concentration was 74.8 (SD (standard deviation) = 23.7) nmol/L in persons born in Norway, which was higher (*p* < 0.001) than those born in Pakistan. None of the Norwegian-born had 25(OH)D levels below 12.5 nmol/L, whereas 9% and 21% of Pakistani men and women had 25(OH)D below 12.5 nmol/L, respectively. However, ethnicity was not defined in the Norwegian-born population. The cross-sectional study of Schleicher et al. [22] assessed vitamin D status of different ethnicities aged 12 years and above (including Mexican American, non-Hispanic Black, non-Hispanic White) at five different time points (1988–1994, 2001–2002, 2003–2004, 2005–2006, 2007–2008 and 2009–2010). The results showed the non-Hispanic Black population have the lower 25(OH)D concentrations and more people with 25(OH)D level < 30 nmol/L than the Mexican American and non-Hispanic White, another cross-sectional study of van der Meer et al. [24] measured vitamin D status of ethnicities in the Netherlands, and reported that Asian and mid/south Africans had lower 25(OH)D concentration and a higher number of people whose 25(OH)D was < 25 nmol/L compared with other ethnicities. Therefore, the above evidence highlights the potential higher risk of vitamin D deficiency in some ethnic minority populations.

The most common cause of rickets is vitamin D deficiency [25]. There was a high prevalence of rickets in children in industrialised Europe and North America in the 19th and early 20th centuries [26], this situation was reversed by human consumption of a variety of vitamin D fortified foods and use of cod liver oil by the late of 1930s. However, in Europe in the 1950s, for food products except breakfast cereals and margarine, it was forbidden to fortify with vitamin D because of cases of vitamin D toxicity in newborns [26]. Consequently, vitamin D fortified products became less available. Unfortunately, rickets has made a resurgence in Europe [27] and also around the world [8], particularly among Asian and Africa ethnic minorities [8,28]. The study of Robinson et al. [29] defined the demographic and clinical characteristics of rickets in Australia, and reported the most prominent regions of origin were India (37%), Africa (33%), and the Middle East (11%), while only 4% were white Australian children. Furthermore, in an earlier UK survey [30] in the West Midlands, paediatricians identified 24 cases of symptomatic vitamin D deficiency in children (≤5 year) and reported an incidence of rickets of 38 and 95 per 100,000 per annum in South Asian and Black children, with only 0.4 per 100,000 per annum in white children.

### 2.3. Vitamin D Status in Different Seasons of Ethnic Minority Populations

Season of the year influences vitamin D status [31]. The study of Hypponen and Power [27] showed that the prevalence of hypovitaminosis D in the UK was especially high in the winter and spring seasons [27]. There are a few studies [10,20,21,23] which reported vitamin D status in different ethnicities in winter. For example Nerhus et al. [20] reported that the ethnic minority population had significantly lower serum 25(OH)D concentrations (mean 29.5 nmol/L, SD = 16.3) in winter than participants from the majority of the population (mean 50.4 nmol/L, SD = 19.1) in Norway, although serum 25(OH)D of different ethnic minorities was not investigated separately. In addition, the study of Adebayo et al. [10] reported ethnic differences in serum 25(OH)D concentration in winter in Southern Finland (60° N), with the mean serum 25(OH)D concentrations in Finnish women (mean 60.5 nmol/L, SD = 16.6) being significantly higher than in East African women (mean 51.5 nmol/L, SD = 15.4). In the UK, the study of Tripkovic et al. [23] reported South Asian participants had much lower serum 25(OH)D concentrations (mean 27.7 nmol/L) than White European participants (mean 60.3 nmol/L) in winter. More specifically, Sacheck et al. [21] compared the serum 25(OH)D values of children in winter across different ethnicities (White, Black, Hispanic or Latino, Asian) in the US. The results showed white children had significantly higher mean serum 25(OH)D concentrations (mean 61.9 nmol/L) than all other children (44.7 nmol/L, 51.9 nmol.L and 46.9 nmol/L for Black, Hispanic or Latino, Asian, respectively). Furthermore, Haggarty et al. [18] investigated the influence of seasonal changes on plasma 25(OH)D in pregnant Caucasian women in Scotland. They found that the highest 25(OH)D values were in summer (53.1 nmol/L, 95% CI (confidence intervals): 50.0, 56.7), and the lowest in winter (34.4 nmol/L, 95% CI: 31.8, 37.2). Also, the greatest proportion of participants whose plasma 25(OH)D was < 25 nmol/L was observed in winter. Another UK study by Darling et al. [14] which compared serum 25(OH)D.

The results showed serum 25(OH)D concentrations of Asians were lower than Caucasians throughout the year with the proportion of participants whose serum 25(OH)D < 25 nmol/L was much higher in Asians (53.5–80.8%) than Caucasians (0.8–10.0%). In addition, the study reported a lack of seasonal changes on 25(OH)D concentrations in the Asian population. Furthermore, vitamin D status are associated with other factors (such as: age, gender, higher latitude, obesity and socioeconomic status) [5], but there are limited evidence of the effects of those influencing factors for the different ethnic minority populations compared with the majority populations, which needs further research to clarity.

### 2.4. Vitamin D Intake of Ethnic Minority Populations

The work of Kiely and Black indicated that dietary vitamin D intakes are inadequate to meet Dietary Reference Intake, which may vary according to gender, age and country fortification practices [32]. Whilst it is known that dietary patterns vary between different ethnic minority populations [33], there is very limited evidence on the vitamin D intake of different ethnicities. Dietary vitamin D intake was reported to be higher in the US and Canada than most of European countries except Nordic countries, due to mandatory fortification in North America [32]. In Finland where mandatory fortification takes place [34], the study of Adebayo et al. [10] showed a higher mean dietary vitamin D intake by women of East African origin (11.2 µg/d, SD = 5.8) than Finish participants (8.4 µg/d, SD = 4.1). The main contributory food sources for dietary vitamin D intake for both East African and Finish participants were fortified fluid milk products and fortified fat spreads. There was a higher intake of fortified fluid milk products in East African group than Finnish group, which may resulted in higher vitamin D dietary intake in the East African group. In the UK, vitamin D fortification of foods is not mandatory [4]. The study of Darling et al. [14] reported that vitamin D intake was slightly higher in Caucasian than Asian sections of the population in the UK throughout the year. Vitamin D intakes from the diet were 1.6–2.2 µg/d and 2.1–2.6 µg/d for South Asians and Caucasians, respectively [14], which was much lower than vitamin D intake of subjects in the Finnish study [10]. In addition, no influence of seasonal changes in dietary vitamin D intake for both South Asians and Caucasians was seen. The main sources of vitamin D in the diet (flour, grains and starches; meat and meat products; fish and fish products, milk and milk products; egg and egg products) were the same for both groups but the proportions of the various foods were different for South Asians and Caucasians. For example, flour, grains and starches contributed 21.8–33.0% and 24.2–26.6% to total vitamin D dietary intake for South Asians and Caucasians, respectively. In the UK National Diet and Nutrition Survey (NDNS) [5], the mean daily vitamin D dietary intake for adults (19–64 years) was 2.8 µg, which was in line with results of Darling et al. [14], however, data in NDNS was not specific analysed for different ethnic minority populations in the UK, which could be done in the future.

### 2.5. Vitamin D Status Response to Vitamin D Supplementation of Ethnic Minority Populations

There is limited evidence on the impact of vitamin D supplementation on the vitamin D status of racially diverse populations (Table 2). The study of Adebayo et al. [10] investigated ethnic differences of serum 25(OH) D to vitamin D_3_ supplementation of 10 or 20 µg/d through a 5-month RCT in East African and Finnish women, and found no ethnic differences in the response to either 10 or 20 µg/d vitamin D_3_ supplementation. In addition, studies of Gallagher et al. [16,17] compared the effect of vitamin D_3_ supplementation at different doses (10, 20, 40, 60, 80, 100 and 120 µg/d) in African American Women with Caucasian women in the US. The findings agreed with Adebayo et al. [10] that effect of vitamin D_3_ supplementation on serum 25(OH)D concentration is not dependent on race.

In contrast, an RCT [21] supplemented different ethnic children (White, Black, Hispanic or Latino, Asian, Multiracial or other) with vitamin D_3_ at three different doses (15, 25 or 50 µg/d) for 6 months. The results showed similar responses across ethnicities for the 15 µg/d and 25 µg/d doses but the Asian group had the lowest response (mean ± SE increase of 35.0 nmol/L ± 5.4 nmol/L) to the 50 µg/d dose group, while Black children had the greatest response to supplementation (mean ± SE increase of 54.4 nmol/L ± 7.0 nmol/L). Furthermore, the study of Aloia et al. [11] used a 6-month RCT to investigate the effect of vitamin D_3_ supplementation on serum 25(OH)D concentration in African Americans and White groups. The results showed both groups achieved the target of 75 nmol/L by week 18, but the vitamin D_3_ dose needed to achieve that value was 50% higher in the African Americans. However, the study of Gallagher et al. [15] found a greater dose response in African Americans than white women after 12-month vitamin D supplementation (up to 60 µg/d). Tripkovic et al. [23] also found a greater response to vitamin D supplementation (15 µg/d for 12 week) in South Asian women than white European women. However, in the studies [11,15,21,23] that reported different effects of vitamin D supplementation in different ethnicities, there was a lower baseline 25(OH)D concentrations in Black and Asian population compared with white people, which may have influenced the different response.

Therefore, future studies on investigating vitamin D status response to vitamin D supplementation for ethnic minority populations need to design the RCT with same serum/plasma 25(OH)D concentration at baseline.

### 2.6. Vitamin D Synthesis from Sunlight Exposure of Ethnic Minority Populations

Skin pigmentation absorbs UVB radiation [35], consequently people with darker skin are susceptible to less UVB absorption. Compared with Caucasians, there is evidence that Asians require approximately threefold longer periods of sunlight exposure because of the protective pigmentation in their skin and Africans need six times the same exposure, to achieve the same serum/plasma 25(OH)D concentration [36]. Furthermore, extensive coverage by garments is practised by some ethnic minority populations due to religious or cultural needs [9], which may add more potential risk of vitamin D deficiency for those ethnic minority populations. In addition there is evidence that some ethnicities may have less sunlight exposure time than Caucasians. Darling et al. [14] showed Caucasians had a significant higher UVB exposure than Asian group (95% CI: 0.3–3.9 SED (standard erythemal dose)) in the UK over the year. Although the reasons could not be assessed in the study of Darling et al. [14], which may be clarified in the future studies.

## 3. Current Strategies and Limitations

Only few foods are naturally rich in vitamin D (e.g., egg yolk, oily fish and wild mushroom), but vitamin D content are highly variable even in those foods considered the richest sources [37]. For example, the study of Mattila et al. [38] measured vitamin D_2_ in different mushroom species, and found that there was a significant variation (0.21–29.82 µg/100 g of fresh weight). For animal derived products, vitamin D concentrations may vary between different produced systems. For instance, wild salmon had nearly double the vitamin D content of farmed salmon [39]; vitamin D_3_ in organic and free range eggs was significantly higher than in indoor eggs [40]. It is therefore difficult to meet the vitamin D dietary recommendation solely by natural foods. Food fortification is a potentially effective strategy to increase vitamin D intake and circulating plasma/serum 25(OH)D concentrations on a population-wide basis. The recent meta-analysis [41] evaluated evidence from sixteen studies and showed a mean individual intake of 11 µg/d from fortified foods (range 3–25 µg/d) increased plasma/serum 25(OH)D concentration by 19.4 nmol/L (95% CI: 13.9, 24.9), which confirmed the efficacy of vitamin D fortified foods on circulating concentrations of 25(OH)D. Currently, however, food fortification policy varies between different countries [30]. For instance, there is mandatory fortification of milk with vitamin D in Canada and Finland, while milk is mostly voluntarily fortified in the US [30,34]. Furthermore, to our knowledge, no studies have investigated the effect of different vitamin D fortified foods on increasing vitamin D status for ethnic minority populations. The review by Cashman et al. [34] suggested that vitamin D should be fortified in a wider range of foods, not only a single staple, to accommodate dietary diversity. This conclusion is especially important to different ethnicities to ensure an adequate vitamin D dietary intake. For example, milk is not widely consumed in India, Jordan or China [42], fortification of wheat flour may be therefore more efficacious in preventing vitamin D deficiency [43]. Therefore, more studies are needed to investigate the effect of vitamin D fortified foods on vitamin D status and human health, especially for the high risk group, such as ethnic minority populations.

## 4. Conclusions

Dark skinned ethnic minority populations generally have a lower vitamin D status than the majority of the population. The main contributory food sources for dietary vitamin D intake were different for ethnic minority populations and majority populations, due to different dietary pattens. Future strategies to increase dietary vitamin D intake by food fortification needs to be explored, specifically for ethnic minority populations. In addition, public health policy and practice needs to have an increased awareness of vitamin D deficiency in ethnic minority populations, and address the dietary strategies for those population in the future.

## Figures and Tables

**Table 1:** Summary of studies investigating the vitamin D status (25(OH)D concentration) in ethnic minority populations (in alphabetic order).

Study/Country	Study Design	Ethnic Minority Population ^a^, *n*	Study Participants, Age, BMI	Season	Vitamin D Intake (µg/day)		25(OH)D Concentration (nmol/L)	
					Mean	SD/95% CI	Mean	SD/95% CI
Adebayo et al., 2018/Finland [10]	Randomised controlled trial	East African, *n* = 47	Women, 41 years, 29.4 kg/m^2^	Winter	11.3	5.1	52.2	14.0
		Finnish, *n* = 69	Women, 33 years, 23.8 kg/m^2^	Winter	8.4	4.1	60.5	16.6
Aloia et al. 2008/US [11]	Randomised controlled trial	Black, *n* = 62	Men and women, 18–65 years, 27.3 kg/m^2^		2.0	NA	39.7	NA
		White, *n* = 76	Men and women, 18–65 years, 26.8 kg/m^2^		2.1	NA	5738.0	Reference
Black et al., 2014/Australia [12]	Prospective cohort study (Western Australian Preganancy Cohort Study)	Caucasian (classified if both parents were Caucasian), *n* = 887	Male and Female, 14–17 year, 21.4–23.0 kg/m^2^	All seasons				
		Non-Caucasian (classified if at least one parent was of an alternate ethnicity), *n* = 158	Male and Female, 14–17 years, 21.4–23.0 kg/m^2^	All seasons			−15.2	−19.1, −11.3 ^b^
Cauley et al., 2011/US [13]	Case control study nested witthin the prospecitve cohort sudy of WHI-OS	White, *n* = 780	Postmenopausal women, 66 years, 27.5 kg/m^2^	All seasons			60.8	24.0
		Black, *n* = 758	Postmenopausal women, 62 years, 30.5 kg/m^2^	All seasons			43.7	21.5
		Hispanic, *n* = 382	Postmenopausal women, 63 years, 29.0 kg/m^2^	All seasons			53.0	21.0
		Asian, *n* = 224	Postmenopausal women, 65 years, 24.7 kg/m^2^	All seasons			62.3	24.3
		American Indian, *n* = 88	Postmenopausal women, 63 years, 29.5 kg/m^2^	All seasons			50.0	25.5
Darling et al., 2013/UK [14]	Longitudinal study	Caucasian, *n* = 128	Premenopausal women, 38 years, 26 kg/m^2^	Summer	2.4	2.0	72.1	26.1
		South Asian, *n* = 43		Summer	2.2	1.8	26.2	9.9
		Caucasian, *n* = 97		Autumn	2.1	1.5	59.5	25.6
		South Asian, *n* = 24		Autumn	2.0	1.4	20.9	11.8
		Caucasian, *n* = 80		Winter	2.6	1.8	44.5	18.0
		South Asian, *n* = 26		Winter	2.0	2.0	19.7	10.6
Gallagher et al., 2012 and 2013/US [16,17]	Randomised controlled trial	Caucasian, *n* = 79		Spring	2.5	1.9	53.2	23.9
		South Asian, *n* = 24		Spring	1.6	1.1	22.1	11.3
		Black, *n* = 110	Women, 67 years, 32.7 kg/m^2^	All seasons			33.0	NA
Gallagher et al., 2014/US [15]	Randomised controlled trial	White, *n* = 163	Women, 67 years, 30.2 kg/m^2^	All seasons			39.0	NA
		Black, *n* = 79	Women, 35 years, 32.5 kg/m^2^	All seasons			37.4	30.7, 43.9
		White, *n* = 119	Women, 33 years, 28.8 kg/m^2^	All seasons			31.0	23.0, 39.2
Haggarty et al., 2013/UK [18]	Prospective cohort study	Caucasians, *n* = 1205	Pregnant women, 31 years, NA	Winter	3.7	3.5, 3.9	34.4	31.8, 37.2
				Spring	3.8	3.6, 4.1	39.7	36.7, 42.9
				Summer	3.9	3.6, 4.2	53.1	50.0, 56.7
				Autumn	4.0	3.7, 4.4	33.7	30.6, 37.2
		Non-Caucasians (African, Asian and Indian), *n* = 42	Pregnant women, 39 years, NA	All seasons			17.1	NA
Meyer et al., 2004/Norway [19]	Prospective cohort study (Oslo Health Study)	Born in Norway, *n* = 866	Men and women, adults, NA	All seasons			74.8	23.7
		Born in Pakistan, *n* = 176	Men and women, adults, NA	All seasons			25.0	13.6
Nerhus et al., 2015/Norway [20]	Prospective cohort study (Thematically Organized Psychosis Study)	Ethnic minority (Turkey, Africa and Latin-America), *n* = 40	Men and women, 28 years, 26.1 kg/m^2^	Winter			29.5	16.3
		Norwegians, *n* = 102	Men and women, 28 years, 24.6 kg/m^2^	Winter			50.4	19.1
Schleicher et al., 2016/US [22]	Cross-sectional: NHANES (2009-2010)	Mexican American, *n* = 1388	Men and women, ≥12 years, NA	All seasons			53.9	52.2, 55.5
		Non-hispanic Black, *n* = 1229	Men and women, ≥12 years, NA	All seasons			46.0	41.6, 50.5
		Non-hispanic White, *n* = 3174	Men and women, ≥12 years, NA	All seasons			75.0	72.5, 77.4
Sacheck et al., 2017/US [21]	Randomised controlled trial	White, *n* = 244	Boy and girl, 11 years, 21.5 kg/m^2^	Winter			61.9	NA
		Black, *n* = 85		Winter			44.7	NA
		Hispanic or Latino, *n* = 135		Winter			51.9	NA
		Asian, *n* = 53		Winter			46.9	NA
Tripkovic et al., 2017/UK [23]	Randomised controlled trial	South Asian, *n* = 90	Women, 43 years, 24.0 kg/m^2^	Winter			27.7	NA
		White, *n* = 245					60.3	NA
van der Meer et al., 2008/The Netherlands [24]	Cross-sectional	Lightest skin Western, *n* = 110	Men and women, 18–65 years, 25.3–28.7 kg/m^2^	All seasons			58.0	49.0, 68.0
		Turkish and North African, *n* = 223	Men and women, 18–65 years, 25.3–28.8 kg/m^2^	All seasons			33.0	28.0, 39.0
		Asian and Mid/South African (darkest skin types), *n* = 280	Men and women, 18–65 years, 25.3–28.9 kg/m^2^	All seasons			29.0	25.0, 34.0

NA: not available; CI: Confidence Intervals; SD: Standard Deviation; NHANES: National Health and Examination Survey; WHI-OS: Women’s Health Initiative- Observational Study. ^a^ Ethnic minority populations refer to populations within a community which has different national or cultural traditions from the majority population, and with darker skin. ^b^ Estimated difference in serum 25(OH)D concentrations from the reference category of categorical variables or per unit increase of continuous variables.

**Table 2:** Summary of randomised controlled trials investigating vitamin D status (25(OH)D concentration) response to vitamin D supplementation in ethnic minority population (in alphabetic order).

Study/Country	Study Duration	Ethnic Minority Population ^a^, *n*	Study Participants, Age, BMI	Season	Vitamin D Supplementation	25(OH) D Concentration nmol/L			
						Baseline		Endpoint	
						Mean	SD/95% CI	Mean	SD/95% CI
Adebayo et al. 2018/Finland [10]	5-month	East African, *n* = 47	Women, 41 years, 29.4 kg/m^2^	Winter	10 µg/ day	52.2	14.0	+10.0	+19.2%
					20 µg/ day			+17.1	+32.7%
		Finnish, *n* = 69	Women, 33 years, 23.8 kg/m^2^	Winter	10 µg/ day	60.5	16.6	+8.5	+14.1%
					20 µg/ day			+10.7	+17.7%
Aloia et al. 2008/US [11]	6-month	Black, *n* = 62	Men and women, 18–65 years, 27.3 kg/m^2^	Winter	97.9 (21.0) µg over 3 visits	39.7	NA		
		White, *n* = 76	Men and women, 18–65 years, 26.8 kg/m^2^	Winter	76.0 (28.4) µg over 3 visits	57.8	NA		
Gallagher et al. 2012 & 2013/US [16,17]	1-year	Black, *n* = 110	Women, 67 years, 32.7 kg/m^2^	All seasons	10–120 µg/day	33.0	NA	125.0	NA
		White, *n* = 163	Women, 67 years, 30.2 kg/m^2^	All seasons	10–120 µg/ day	39.0	NA	117	NA
Gallagher et al. 2014/US [15]	1-year	Black, *n* = 79	Women, 35 years, 32.5 kg/m^2^	All seasons	60 µg/ day	37.4	30.7, 43.9	97.6	90.4, 104.8
		White, *n* = 119	Women, 33 years, 28.8 kg/m^2^	All seasons		31.0	23.0, 39.2	107.8	95.4, 120.1
Sacheck et al. 2017/US [21]	1-year	White, *n* = 244	Boy and girl, 11 years, 21.5 kg/m^2^	Winter	50 µg/ day	61.9	NA		
		Black, *n* = 85		Winter	50 µg/ day	44.7	NA	+54.4	7.0
		Hispanic or Latino, n = 135		Winter	50 µg/ day	51.9	NA		
		Asian, *n* = 53		Winter	50 µg/ day	46.9	NA	+35.0	5.4
Tripkovic et al. 2017/UK [23]	12-week	South Asian, *n* = 90	Women, 43 years, 24.0 kg/m^2^	Winter		27.7	NA	60.1	49.7, 70.5 ^b^
		White, *n* = 245				60.3	NA	87.9	82.3, 93.5 ^b^

NA: not available; BMI: Body Mass Index; ^a^ Ethnic minority populations refer to populations within a community which has different national or cultural traditions from the majority population, and with darker skin; ^b^ Juice supplemented with 15 µg vitamin D_3_.
